# Anterior tibial artery pseudoaneurysm as a rare technical complication after corrective fibular osteotomy: a case report

**DOI:** 10.1186/s13037-022-00334-9

**Published:** 2022-07-30

**Authors:** Yu-Sheng Chen, Chyun-Yu Yang, Chih-Wei Chang, Yen-Nien Chen

**Affiliations:** 1 Department of Orthopedics, National Cheng Kung University Hospital, College of Medicine, National Cheng Kung University, Tainan, Taiwan; 2 Department of Orthopedics, Kuo General Hospital, Tainan, Taiwan; 3 Department of Orthopedics, College of Medicine, National Cheng Kung University, Tainan, Taiwan; 4 Department of Physical Therapy, Asia University, Taichung, Taiwan

**Keywords:** Knee OA, Partial fibular osteotomy, Anterior tibial artery, Pseudoaneurysm, Complication

## Abstract

**Introduction:**

Partial fibular osteotomy has been recognized as a surgical alternative to treat medial compartment osteoarthritis of the knee. Related peroneal neuropathies are of concern among the relatively few complications after this procedure. In our clinical practice, the osteotomy level has therefore been modified to avoid the above defects. However, a rare case of vascular injury was encountered. Herein we describe an accidental anterior tibial artery pseudoaneurysm as a rare technical complication after this corrective osteotomy.

**Case presentation:**

A 55-year-old male visited our emergency room, presenting a painful swelling over his right anterolateral shin along with surrounding ecchymosis. Thirteen days ago, he just underwent a corrective fibular osteotomy over his right painful varus knee at our institute, and was discharged after an uneventful postoperative stay. Urgent angiography revealed an out-pouching vascular lesion, pseudoaneurysm, involving his right anterior tibial artery. Prompt endovascular repair with stent insertion and balloon compression successfully stopped the persistent extravasation from the injured artery. Follow-up angiography as well as outpatient review confirmed the regression of this lesion and associated symptoms without sequelae.

**Conclusion:**

Although corrective fibular osteotomy is a simple surgical procedure, it is not free of complications. The suggested osteotomized level in the pertinent literature predisposes patients to certain neuromuscular deficits, which could be avoided by the modified level of osteotomy. However, our case highlights surgeons’ familiarity with certain risky neurovascular structures around the osteotomy site and corresponding technical considerations. A recent surgical history along with alarming symptoms/signs should arouse clinical suspicion, aid in timely identification and make appropriate interventions for potential vascular complications.

## Introduction

Recently, researchers observed the decompression of the medial knee joint following partial fibular osteotomy and proposed it as a surgical alternative to other surgical modalities for medial compartment knee osteoarthritis (OA) [1–3]. However, this surgery was not free of complications at all [2, 4], and the most commonly reported complication is related to insults to the peroneal nerve and its branches [4]. In contrast, related vascular events have rarely been documented in the pertinent literature. Herein, we describe a rare case of delayed anterior tibial artery pseudoaneurysm following corrective fibular osteotomy, successfully managed with endovascular repair.

## Case report

**Fig. 1:**
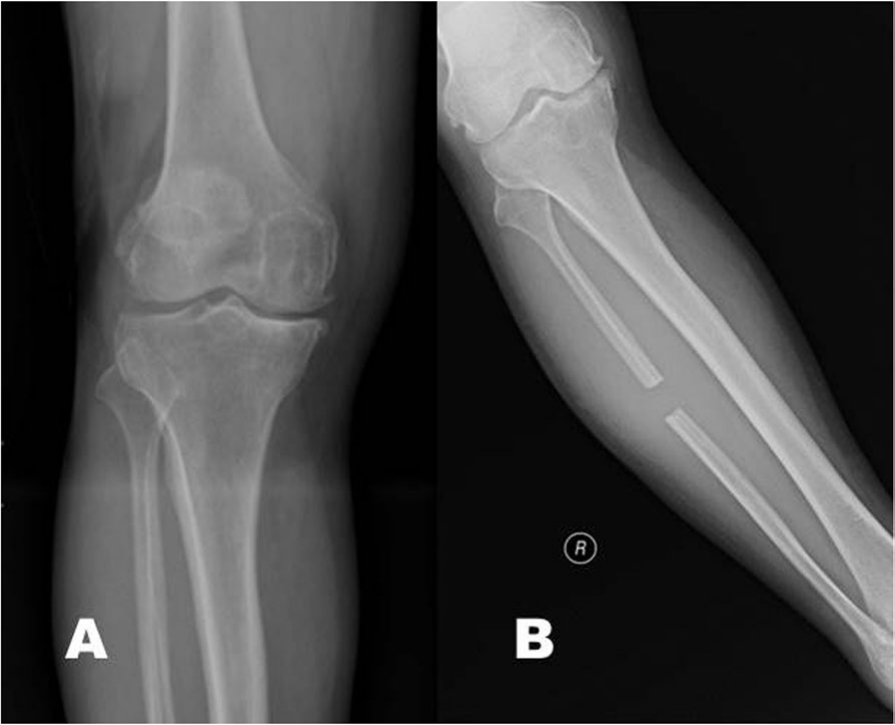
**A** Preoperative anterior–posterior x-ray. **B** Postoperative anterior–posterior x-ray: modified partial fibular osteotomy at the level of 15 cm below the fibular head

A 55-year-old male recycle worker (height, 164 cm; weight, 79 kg) with underlying asthma and chronic hepatitis B suffered his right knee pain for years. Upon radiographs and related clinical presentations, right medial compartment knee OA was diagnosed (Fig. 1A). Due to his relatively young age and working demand, partial fibular osteotomy was his preferred intervention over common knee arthroplasties or corrective tibial osteotomy. Written informed consent was obtained prior to the surgery.

**Fig. 2:**
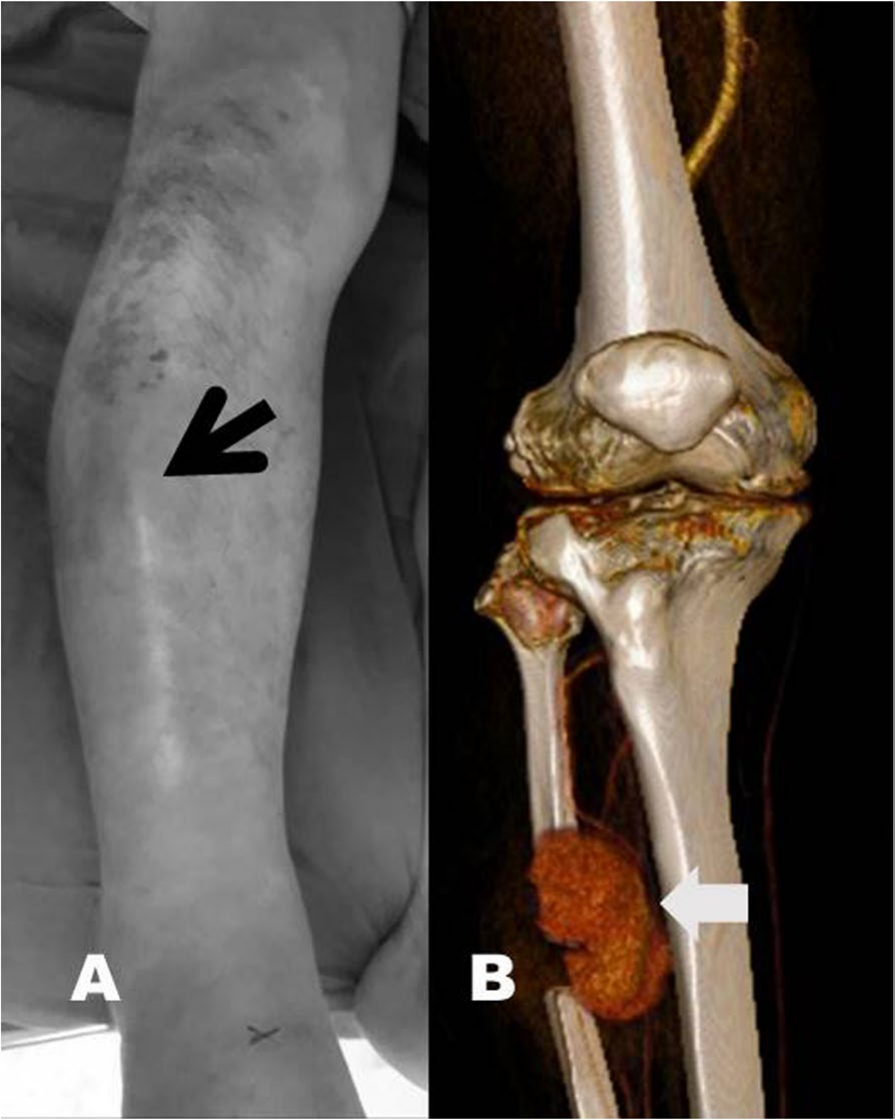
**A** Clinical photo at emergency room; surrounding ecchymosis and swelling (black arrow). **B** Reconstructed computed tomography angiography; an out-pouching psuedoaneurysm (white arrow) aries from the anterior tibial artery

**Fig. 3:**
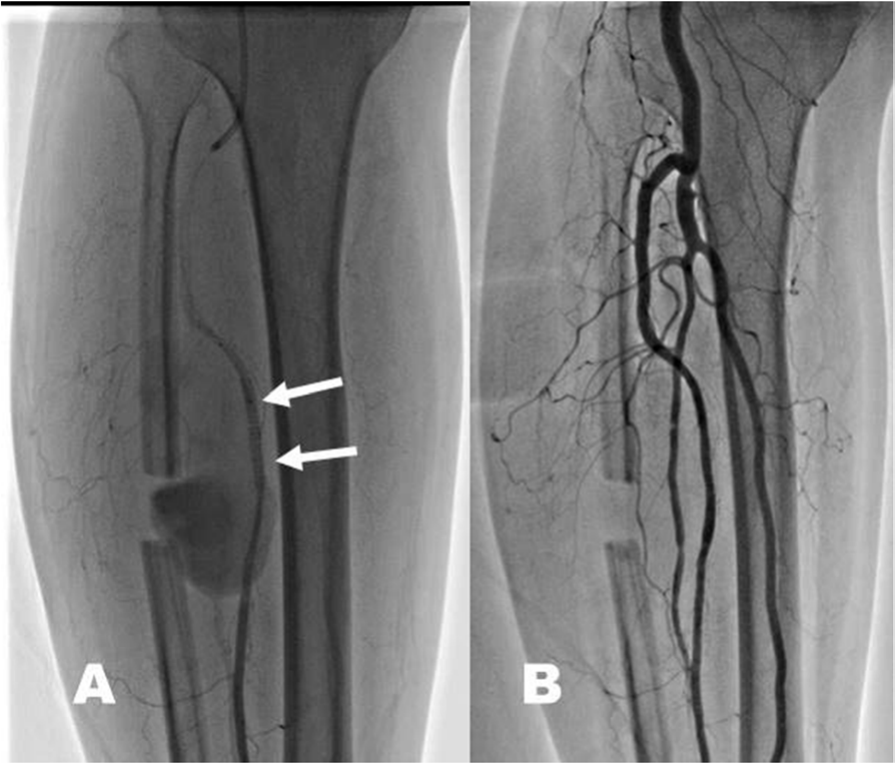
**A** Endovascular repair with insertion of two stent grafts (white arrows). **B** Follow-up angiography 2 days later; patent blood flows within the right lower leg without active extravasation from the anterior tibial artery

The index procedure was performed under the lateral decubitus position with slight knee flexion and tourniquet control. To expose the fibula, a posterolateral approach passing between the peroneus longus muscle and the soleus muscles was executed in an 8-cm skin incision. A 2-cm bone segment was removed from the fibula at a level of 15 cm below the fibular head (Fig. 1B), and comprehensive hemostasis was executed after the release of the pneumatic tourniquet. During the 2-day inpatient stay, no evident painful swelling developed, and he was discharged with independent ambulation. However, he returned to our ER on postoperative day 13, presenting with local heat, tenderness, swelling and ecchymosis around the surgical site (Fig. 2A). Computed tomography angiography (CTA) showed an out-pouching vascular lesion, pseudoaneurysm, involving his right anterior tibial artery (Fig. 2B). Due to persistent extravasation and symptoms, endovascular repair was performed by the intervention radiologist using two stent grafts (3.5 mm/19 mm and 2.8 mm/19 mm stents; Graftmaster stent graft system®, Abbott, USA) along with a 3.5 mm balloon catheter (Fig. 3A). Two days later, the follow-up angiography confirmed the patent blood flow within his right anterior tibial artery without active bleeding (Fig. 3B). Subsequent anticoagulants (cilostazol 50 mg/tab orally twice a day; clopidogrel 75 mg/tab orally one dose per day) were prescribed to prevent stent occlusion. At his 3-month outpatient review, the patient was able to walk independently with little knee pain, and his shin painful swelling had regressed without sequelae.

## Discussion

Knee osteoarthritis is a common disease in the elderly that causes painful ambulation, motion limitation and ultimately disability [5, 6]. In 2015, Yang et al. suggested that fibular osteotomy could relieve pain and improve the varus alignment of medial compartment knee OA by redistributing loads between the medial and lateral compartments [2]. The authors proposed that partial fibular osteotomy is a simpler, less invasive and cost-effective intervention compared to other common surgeries. A further biomechanical cadaveric study in 2018 proved decompression of the medial compartment after proximal fibular osteotomy intervention [1].

In the early literature (mainly from 2015 to 2018), a relatively low rate of complications was mentioned, and most of them occurred as peroneal neuropathies due to their proximity and an anatomical course about the fibula. The original authors reported 3.6% lower leg numbness due to common peroneal nerve palsy and superficial peroneal nerve injury [2]. They also reported that 14.5% of cases with transient leg weakness recovered in 4 weeks. Even a higher rate of neuromuscular complications, such as extensor hallucis longus weakness and paraesthesia over dorsolateral foot and anterolateral aspect has been documented by some Indian researchers [7, 8], which was comparable with our early experiences in partial fibular osteotomy based on the suggested osteotomized level (6–10 cm below the fibular head) in most literature [2, 4].

Ogbemudia AO [9] reported a reduced incidence of peroneal nerve involvement with distal fibular resection compared to proximal fibular resection. To avoid troublesome drop hallux and paraesthesia foot, we thus moved the fibular osteotomy downward to a more distal level (10–15 cm below the fibular head) in our later practice. No more neurologic compromise but a similar effect in pain relief was obtained until the rare case of vascular complication.

Due to the different osteotomized level in our case and the few complications mentioned in prior literature, we searched for reported vascular injuries following the harvest of fibular grafts, a similar surgery to proximal fibular osteotomy removing a fibular segment as bone grafts. Related complications to fibular harvest consist of nerve injury (3 ~ 12%), compartment syndrome, weakness of the extensor hallucis longus (3 ~ 10%), and ankle instability (2–12%) [10–13] while vascular complications are relatively rare with a prevalence less than 1%. Among the vascular complications, some were thromboembolic events, some intraoperative vascular injuries were repaired immediately, and no delayed presentation was mentioned [11].

In the literature, the incidence of anterior tibial artery pseudoaneurysms is relatively low, and common associated events are trauma, vascular and orthopaedic procedures. Reported causative orthopedic procedures included the insertion of an interlocking bolt in tibial nailing and Steinman pin insertion while performing skeletal traction [14]. Rupp et al. [10] studied the danger zones while performing fibular osteotomy and reported that the peroneal nerve and its muscular branches are at primary risk at the proximal one-third of the fibula. Anatomically, the anterior tibial artery is most vulnerable while penetrating through the interosseous membrane at approximately 5.4 cmbelow the fibular head to the anterior compartment [10]. Then it runs down anterior to the interosseous membrane medial to the fibula in the anterior compartment [15]. To secure vulnerable neurovascular structures of the middle to upper third of the fibula, a posterolateral approach between the peroneus longus and the soleus muscles and fibular osteotomy aiming toward the anterior tibia were recommended. Considering the relatively safe approach we applied to all our patients, a smooth postoperative inpatient stay and the delayed presentation of this vascular lesion, it is difficult to determine the actual timing of this unexpected insult. However, the sharp bony edge on the flabby fibular stump always endangers the corresponding vessels nearby. Blunting the sharp bone edges after osteotomy, gentle soft tissue handling while stripping from the bone and careful protection as oscillating saws proceed forwards should be considered mandatory to avoid iatrogenic neurovascular injuries in all such procedures.

Limb pseudoaneurysms frequently present with pain, swelling, ecchymosis, pulsating masses and compressive neurological symptoms. However, it may take hours to years to be clinically symptomatic depending on the size and site of the pseudoaneurysm [15, 16]. Therefore, once the mentioned vascular symptoms become prominent, any history of nearby surgeries should raise clinical suspicion and warrant prompt investigation.

Currently, this is the only case report that describes an anterior tibial artery pseudoaneurysm after partial fibula osteotomy that was successfully managed with endovascular stenting. The presentation of painful swelling and ecchymosis at nearby surgical sites are alarming signs indicating vascular imaging, such as CT angiography for potential vascular injuries. Referral to interventional radiologists or vascular surgical teams should be done promptly.

## Conclusion

Although corrective fibular osteotomy is a simple surgical procedure, it is not free of complications. The suggested osteotomized level in the pertinent literature predisposes to peroneal neuropathy and even muscle weakness, which could be avoided by the modified level of osteotomy. However, our case highlights surgeons’ familiarity with certain risky neurovascular structures around the osteotomy site and corresponding technical considerations. A recent surgical history along with alarming symptoms/signs should arouse clinical suspicion, aid in timely identification and make appropriate interventions for potential vascular complications.

## Data Availability

All materials (consent, pictures, labs) are available for review if needed.

## Abbreviations

CTA: Computed tomography angiography
OA: Osteoarthritis
